# Qualitative analyses on the classification model of bystander behavior in cyberbullying

**DOI:** 10.3389/fpsyg.2023.1152331

**Published:** 2023-07-11

**Authors:** Kexin Rong, Xiaowei Chu, Yujing Zhao

**Affiliations:** ^1^School of Psychology, Zhejiang Normal University, Jinhua, China; ^2^Intelligent Laboratory of Zhejiang Province in Mental Health and Crisis Intervention for Children and Adolescents, Jinhua, China

**Keywords:** cyberbullying, bystander, behavior intention, actual behavioral response, classification, qualitative study

## Abstract

**Introduction:**

Bystanders account for the largest proportion of those involve in cyberbullying and play an important role in the development of cyberbullying incidents. Regarding the classification of bystander behavior in cyberbullying, there exist some limitations in the previous research, such as not considering the complexity of the online environment. Therefore, this study constructed a new classification model of bystander behavior in cyberbullying.

**Methods:**

By separately utilizing questionnaires and experimental methods, the study collected participants’ behavioral intentions and actual behavioral responses to deal with cyberbullying incidents.

**Results:**

Based on two qualitative studies, this study summarized a new classification model, which included three first-level factors and six second-level factors. Specifically, the classification model included positive bystander behavior (i.e., pointing at the victim, bully, and others), neutral bystander behavior (i.e., inaction), and negative bystander behavior (i.e., supporting and excessively confronting the bully).

**Discussion:**

The classification model has important contributions to the research on bystander behavior in cyberbullying. This model helps researchers to develop more effective intervention approaches on cyberbullying from the perspective of each category of bystander behavior.

## Introduction

Cyberbullying is a prevalent social phenomenon. As people have more access to the Internet, the rate of cyberbullying is rising (Kowalski et al., 2019). Brochado et al. (2016) summarized that the prevalence of cyberbullying ranged from 11.2 to 56.9% in China. Numerous studies showed that cyberbullying had diverse negative effects on the victim (e.g., anxiety, substance use, and suicidal ideation; Kim et al., 2018; Zhu et al., 2021). The previous research on cyberbullying has been mostly conducted from the perspective of the bully and victim. Nevertheless, a large number of bystanders exist in cyberbullying incidents. For example, Duggan (2017) found that more than 65% of Internet users have witnessed cyberbullying. Bystanders also play an essential role in preventing cyberbullying incidents. Like the Butterfly Effect, a bystander’s actions may trigger the reactions of others, which may change the entire event. Many studies confirmed that bystanders’ different behavioral responses had a significant impact on the behavior of the bully, the victim, and other bystanders (Bastiaensens et al., 2014; Leung et al., 2018), thus affecting the development process of the whole cyberbullying incident. For instance, Leung et al. (2018) found that when bystanders gave positive feedback to the bully through such behaviors as forwarding, the hurtful information that the bully had posted would be spread more widely on the network.

### Previous classifications of bystander behavior in cyberbullying

In recent years, researchers have classified bystanders’ behavior in cyberbullying into different categories. First, scholars divided bystander behavior into *two categories* (Bastiaensens et al., 2014; Olenik-Shemesh et al., 2015; DeSmet et al., 2016). For example, DeSmet et al. (2016) considered that bystander behavior included positive (e.g., defending or comforting the victim) and negative bystander behavior (e.g., joining and assisting the bully). Bastiaensens et al. (2014) divided bystanders’ behavioral intentions into helping the victim and reinforcing the bully. Olenik-Shemesh et al. (2015) distinguished bystanders’ active and passive behavior based on whether they provided help to victims. The two categories of bystander behavior that researchers classified are similar to bystanders’ prosocial and antisocial behaviors in cyberbullying.

Some scholars also divided bystander behavior in cyberbullying into *three categories* (DeSmet et al., 2012; Wachs, 2012; Van Cleemput et al., 2014). For example, DeSmet et al. (2012) divided bystander behavior into reinforcing the bully, defending the victim, and passive bystanding. Wachs (2012) divided bystander behavior into three categories: assisting and reinforcing (i.e., providing positive feedback to the bully to continue the cyberbullying), defending (i.e., helping the victim), and bystanding (i.e., doing nothing). Van Cleemput et al. (2014) focused on joining in bullying, helping the victim, and doing nothing. The three categories are developed based on the two categories, with adding passive bystanding as the third category of bystander behavior.

Many researchers classified bystander behavior in cyberbullying into *four categories* (Thornberg and Jungert, 2013; Dillon and Bushman, 2015; Song and Oh, 2018). For instance, Song and Oh (2018) believed that bystanders who witnessed cyberbullying could play four types of roles: defender, assistant, reinforcer, and outsider. Besides, Thornberg and Jungert (2013) considered that bystanders could play the four roles above. Other researchers believed that when bystanders faced emergencies or violent events online, they had four behavioral choices, including direct intervention, indirect intervention, joining in the bully, and inaction (Dillon and Bushman, 2015). Direct intervention was the behavior in which bystanders provided help to the victim by successfully going through the five steps of the Bystander Intervention Model (Latané and Darley, 1970). Indirect intervention, or detour interventions, might involve more micro-decisions, such as reporting emergencies to authorities (Dillon and Bushman, 2015). The four categories are further detailed based on the three categories, with one of them being more subdivided.

Fewer researchers divided the bystander’s behavior in cyberbullying into *five categories* (Jones, 2014). Jones (2014) created a type of cyberbullying bystander based on bystanders’ purpose and behavior. The roles of bystanders included the oblivious/distant bystander (i.e., ignoring), the entertained bystander (i.e., observing), the conspiring bystander (i.e., intentional instigating, such as assisting the bully), the unintentional instigating bystander (e.g., confronting the bully), and the active/empowered bystander (i.e., intervening, such as reporting the cyberbullying to authorities and responding to the bully directly).

### Limitations in the previous research

The classifications of bystander behavior in cyberbullying in the previous research have three major limitations. The first limitation is that most categories of bystander behavior in cyberbullying are simply transferred from those in traditional bullying. Traditional role classification in bullying was based on self-evaluation behavior, social acceptance and social rejection, and the affiliation of social status groups. In other words, previous classification models do not take into account the complexity of the online bystander’s environment. Compared to bystanders in traditional bullying, the characteristics of the Internet make bystanders in cyberbullying present some new characteristics. First, in cyberbullying incidents, there are less time and space limits, and the permanence of incidents in the network (Whittaker and Kowalski, 2015) may make the number of bystanders increase. Second, information and communication technologies enable bystanders to get more forms of expression, such as “likes” and forwarding, private comments, and public refutations. Third, the openness and anonymity of online platforms enable the transformation of identity in cyberbullying to achieve easily. That is, some bystanders become bullies or victims more easily (Huang et al., 2019).

The second limitation is that the definition of some concepts is vague. Specifically, negative bystander behavior includes reinforcing and assisting the bully. Reinforcement is to give positive feedback to bullies to strengthen their bullying behavior (Quirk and Campbell, 2015). Assistance is the act of joining in the bullying later, though without starting the bullying initially (Pozzoli and Gini, 2010). In traditional bullying, the line between reinforcement and assistance is fairly clear. However, the act between the two sometimes overlaps, and the line is blurred in cyberbullying. For instance, giving an encouraging expression in bystanders’ eyes to the bully is a reinforcement for the bully in traditional bullying, because the origin of bullying is from the original bully. While upvoting or forwarding bullies’ comments in cyberbullying is not only the behavior of reinforcement but also the behavior of assistance, because many people on the Internet support the comments by upvoting or forwarding rather than sending their opinions again when they approve of the bully’s behaviors or views. The direct source of harm for the victim is to see insulting remarks about them. With the number of “likes” and forwarding increasing, the harm for the victim is accumulating. Bystanders’ reinforcing behavior is, in effect, equivalent to assisting the bully, since the origin of bullying is not merely from the original bully but bystanders.

The third limitation is that the categories are too general to include all bystander behavior in cyberbullying. Most previous research classifies first-level factors of bystander behavior in cyberbullying, without further sorting and analyzing the second-level factors. It is not that a greater number of first-level factors is better, but the second-level factors based on the first-level factors could make the classification model more focused. For instance, defending refers to helping the victim and is considered as a positive bystander behavior (Desmet et al., 2012; Wachs, 2012; Van Cleemput et al., 2014). Nevertheless, the researchers do not mention whether confronting the bully is an action to help the victim. In traditional bullying, confronting the bully helps the victim and prevents the bullying from deteriorating, which is positive indeed. However, the situation is more complicated in cyberbullying because improper protection will lead to another cyberbullying. Specifically, bystanders are involved in cyberbullying incidents and become bullies, which will expand the scope of bullying incidents and form a vicious circle. The consequences of such behavior are not positive indeed, which begs the question of whether confronting the bully cannot be classified as positive bystander behavior. The classification of bystander behavior in cyberbullying of similar nature needs to be deliberated.

The fourth limitation is that researchers do not coincide in opinions on the analysis of bystanders’ inaction. Some researchers believed that inaction might be considered as acquiescence and approval of bullying behavior by both the bully and the victim, thus further aggravating the incidents (Kowalski et al., 2014; DeSmet et al., 2016). Some researchers, regarding outsiders as “potential defenders,” considered that outsiders accounted for the majority of bystanders and should be intervened to turn into defenders of the victim (Song and Oh, 2018). Some researchers believed that when bystanders in cyberbullying remained passive and inactive (e.g., do not post or forward comments but remove nasty materials), their behavior could be regarded as positive for the victim. Because the harmful actions were contained (Barlińska et al., 2013).

The fifth limitation is that most classifications are proposed based on researchers’ theoretical speculation and lack empirical evidence, which raises several issues. First, the applicability of classification models proposed based on theory without empirical support is questionable. Second, classification models proposed by researchers according to their research purposes are more one-sided and do not consider all possibilities of bystander behavior. Third, researchers have different classification models, and the results they obtained are more scattered and not systematic and coherent.

### Overview of present analyses

To systematically study bystander behavior, a classification model of bystander behavior in cyberbullying is needed as a theoretical foundation. Through literature review, previous research has identified different forms of classification of bystander behavior: two, three, and four categories. However, the previous classifications are simply transferred from those in traditional bullying, are too general, and lack empirical evidence. This study aims to sort out a classification model of bystander behavior in cyberbullying. To achieve the aim, two qualitative studies are conducted to, respectively, analyzing bystanders’ behavioral intentions and actual behavioral responses. It may develop bystander-based interventions on cyberbullying, which are beneficial for preventing cyberbullying or alleviating the harm on victims.

## Study 1

Following the previous research that measured bystanders’ behavioral intentions in cyberbullying, the present study first conducted a qualitative analysis to investigate participants’ behavioral intentions toward cyberbullying incidents. This is a relatively convenient method to collect the types of participants’ responses as much as possible. In this sense, the classification model of bystander behavior in cyberbullying can be initially proposed.

### Method

#### Participants

The convenience sampling method was adopted to recruit participants. The sample was relatively representative because they were from several colleges in Chinese cities with intermediate levels of economy and education. A total of 448 students participated in the survey. After deleting invalid cases (e.g., the response time was less than 3 min, and response intent was meaningless), 434 valid cases were finally obtained. The age of the valid participants ranged from 18 to 25 years (*n* = 434, *M* = 20.42, SD = 1.84), and 65.4% of them were female.

#### Procedure

The Ethics Committee of the authors’ university approved the present study. The questionnaire in this study was edited and generated in the Questionnaire Star (i.e., a professional and popular platform for editing questionnaires and collecting survey data in China). The website link of the questionnaire was sent to participants, and they were instructed to give their responses online. At the beginning of the survey, participants were required to carefully fill in the questionnaire according to their actual situation. Furthermore, they were informed that the survey was conducted anonymously, there was no right or wrong answer to the questions, and the data of their answers would not be disclosed. After obtaining the informed consent of the participants, their basic information (e.g., gender and age) would be collected in the questionnaire.

Then, a screenshot that simulated the cyberbullying incident was presented. Three persons in the screenshot were simulated, respectively, as being a bully (named “whl”), a victim (named “Cxh”), and a target bystander (named “Kongliu” in Chinese). To control the effect of sex on participants’ responses, their nicknames and avatars were obtained as being the most neutral through previous selection and evaluation. The nicknames and avatars of other bystanders were also balanced across sex. In these screenshots, four common types of cyberbullying were simulated (i.e., deliberately ignoring, teasing, insulting, and revealing privacy), and each type of cyberbullying presented two incidents. In each incident, the victim first shared their daily lives or sought others’ help, and then the bully verbally attacked the victim. The features of the incident (i.e., universality, familiarity, severity, urgency, and specificity) had been previously assessed and controlled. To control the effect of the number of other bystanders in cyberbullying incidents, the situations were set, respectively, for 0, 1, 14, and 49 other bystanders in each incident. The number of other bystanders was manipulated by showing the online number displayed at the top of the chat group. A total of 32 cyberbullying situations with 4 (types of cyberbullying) × 2 (the number of simulated incidents) × 4 (the number of other bystanders) were simulated.

Screenshots of these cyberbullying situations were presented to the participants at random. The participants were informed that the picture was a screenshot of chat content in an online group, and they were asked to carefully watch the chat content and other relevant information in the screenshot. Afterward, they needed to imagine themselves as the “Kongliu” and respond to what they would normally do in the face of such an incident by typing no less than 10 words. At the end of the survey, the money reward ranging from 1 to 5 RMB was randomly given to the participants. The survey was distributed and collected from January 8 to 14, 2020, lasting about 1 week. The average response time for completing the survey was 13.84 min. Detailed information about the research materials and procedures can be seen in Chu’s (2020) study.

#### Data analyses

All survey information was verbatim transcribed and input into Excel 2016 and SPSS 21.0. The whole data were sorted out and analyzed by using Word 2016 and NVivo 11.0. The coding team included an associate professor and two postgraduates majoring in psychology, whose research focused on adolescent cyberpsychology and behavior. The team used the grounded theory approach to analyze the data. In the process of coding, this study followed the principles of “more is better than less” and “allow some repetition, but avoid excessive merging.”

The coding process mainly took four steps: familiarizing with the data, generating the initial codes, discussing and refining, and naming the node. The team inspected all the data and eliminated irrelevant information. For example, the answers from the perspective of the victim were deleted (e.g., “telling the bully that I do not like being teased”). Then the valid data were analyzed qualitatively to generate the third-level initial codes. The coder discussed with team members, analyzed the uncertain data, and interpreted certain data to improve the coding and make it more reasonable and precise. Next, the coder classified and merged the third-level codes and further generated the second-level codes, which in turn, generated the first-level codes step by step. The coder discussed with team members and improved the quality of the whole coding results. Finally, the nodes obtained from the previous coding results were named.

### Results

The results of the qualitative analysis of participants’ behavioral intentions in cyberbullying were displayed in Table 1. Participants’ behavioral intentions included positive, neutral, and negative behavior in cyberbullying. Specifically, positive bystander behavior referred to the actions that were beneficial to the benign development of cyberbullying incidents and alleviate the negative influence of the victim, which included pointing at the victim, bully, and others. Pointing at the victim represented expressing support and offering help to the victim. Pointing at the bully, or confronting the bully moderately, represented stopping cyberbullying incidents and protecting the victim in a rational way for the victim. Pointing at others represented seeking help from other people except for the bully and the victim to prevent cyberbullying incidents. Neutral bystander behavior referred to the behavior with uncertain influence on the development of cyberbullying incidents, including inaction. Negative bystander behavior referred to the behavior that deteriorated and spread the cyberbullying incidents, including supporting the bully and confronting the bully excessively. Supporting the bully represented directly reinforcing, assisting, or joining the bully in the cyberbullying. Confronting the bully excessively represented protecting the victim by irrationally punishing the bully (e.g., abusing and rumoring).

**Table 1:** Participants’ behavioral intentions to cyberbullying incidents.

First-level codes	Second-level codes	Third-level codes	Examples
Positive behavioral intentions (499)	Pointing at the victim (243)	Defending the victim (34)	Everyone has his shortcomings, and he just asks.	Encouraging the victim (24)	Come on! I believe you can do it.	Comforting the victim (36)	Do not worry too much.	Helping the victim in private chat (39)	Provide suggestions to the victim in private chat.	Responding to the victim (79)	Ask if there’s anything I can do for you.	Providing suggestions to the victim (31)	Ignore the bully. Be sensible and be yourself.
	Pointing at the bully/confronting the bully moderately (245)	Chatting with the bully in private (32)	Remind the bully to withdraw the message in private chat.	Expressing the speechlessness of the bully (6)	……….	Telling the bully politely that you think it is wrong (50)	Point out that the bully’s way of speaking is wrong.	Stopping the bully rationally (30)	Communicate rationally, resolve problems peacefully, and act as a peacemaker.	Requiring the bully (28)	No personal attacks.	Providing suggestions to the bully (18)	You do not have to answer if you do not want to.	Persuading the bully (38)	Persuade the bully it’s not as serious as he said.	Placating the bully (2)	Oh, whl. Come on.	Breaking the ice (21)	Crack a joke to ease the situation.	Changing the subjects (20)	Talk about something happy.
	Pointing at others (11)	Finding friends to help (2)	Mobilize people around to stop it.	Reporting to the authority (9)	Ask the administrator to kick the bully out of the chat group.
	Neutral behavioral intentions (89)	Inaction (89)	Keeping silent (42)	Just look at it. I will not say anything.	Disregarding (34)	Close the chat group.	Not knowing what to do (6)	I do not know what to say to mediate the dispute.	Taking a wait-and-see attitude (7)	Let me think about whether to talk in the chat group.
		Negative behavioral intentions (91)	Supporting the bully (21)	Defending the bully (11)	I thought it was a harmless joke.	Watching the scene of bustle (6)	Bravo!	Pointing out the victim’s faults (3)	The victim also did something wrong.	Joining the bully (1)	I scold the victim also. Because it is silly that he cannot use Baidu to solve problems.
			Confronting the bully excessively (70)	Taunting the bully (42)	Who do you think you are?/Take a hike.	Blaming the bully (11)	Denounce the bully’s behavior.	Abusing the bully (15)	Scold the bully until he apologizes.	Posting the bully’s cyberbullying on the Internet (2)	To capture a screenshot and post it on the Internet.

The numbers in brackets indicate the frequency of such behavioral intentions.

The positive behavioral intentions that most participants reported were pointing at the victim and pointing at the bully. The former included responding to the victim and helping the victim in private chat, while the latter included chatting with the bully in private and telling the bully politely that you think it is wrong. The majority of the participants with neutral behavioral intentions directly expressed their choice to keep silent or disregard the cyberbullying incidents. Few of those with neutral behavioral intentions gave reasons for their inaction, such as not knowing the truth of the matter, not seeking to pay attention to it, and not knowing what to do. Participants’ negative behavioral intentions mainly focused on confronting the bully excessively by taunting the bully.

### Discussion

The present study aims to investigate participants’ behavioral intentions toward cyberbullying incidents. The classification model of bystander behavior in cyberbullying can be initially conceived through Study 1. On the whole, most participants show positive behavioral intentions, while the number of neutral and negative behavioral intentions is relatively few. It is possibly influenced by social desirability. The participants are more likely to show the behaviors expected and accepted by the public (Vernon, 1934). The 3 sec-level factors of positive cyberbystander behavior all have the behavioral intention to chat privately. It may be that private chat makes them feel more anonymous and prevents them from being evaluated by others so that evaluation apprehension will not be generated (Latané and Nida, 1981; Fischer et al., 2011). It is also possible that public responses often lead to too many messages back and forth, and sending private messages prevents their responses from being ignored. Therefore, participants tend to prefer the behavioral intention of private chat. Some participants give generalized responses for possible behavior, such as “I will criticize the bully” and “mobilize people around to stop it.” It follows that participants’ sense of substitution may not be strong in the simulated situations. This is probably because they are asked to imagine how they might react as a bystander in cyberbullying just by looking at a static screenshot, without other information.

## Study 2

Study 2 was conducted to measure the bystanders’ actual behavioral responses by using the dynamically scenario-simulated method in the real online environment, which reduced the potential bias caused by social desirability, and also made up for the lack of sense of immersion and substitution in Study 1. Specifically, Study 2 collected the data on bystanders’ actual actions in six types of real online groups, in which eight cyberbullying incidents were dynamically simulated. The scope of the participants was not limited to college students. Study 2 tested the preliminary classification model in Study 1, which made the model more applicable.

### Method

#### Participants

The experimenters joined 230 QQ chat groups involving an average of 74,729 people online. The groups contained six themes (i.e., fan gossip, study and examination, online games, film and television entertainment, making friends in the same city, and life of leisure). A total of 231 users spoke in the groups after presenting cyberbullying incidents. Considering the users’ anonymity online, their personal information on the homepages may not be detailed or real, hence their sociodemographic information (e.g., gender and age) could not be collected.

#### Procedure

Each experimenter applied for two new QQ accounts. One represented the bully named “whl,” and the other represented the victim named “Cxh.” The avatars of the two simulated users were randomly chosen from the neutral avatars, which had been previously assessed across sex (see Study 1). To control the potential effects of sociodemographic factors on the participants’ responses, the two users did not set their sociodemographic information (e.g., gender, age, status, and location) on the QQ homepage (or make-up false information).

The whole procedure of the experiment included three steps. First, the experimenters applied to join six types of QQ groups. They successfully joined at least ten groups under each condition of different numbers of online users (< 5, 6–20, 21–50, 51–100, 101–200, 200–500, 501–1,000, and > 1,000), with a total of 230 groups. This is to control the possible effect of the number of bystanders on the research results. Second, the experimenters presented the eight cyberbullying incidents an average of two times in six types of online groups. The bully noticed the victim by using the @ symbol to avoid others’ misunderstanding of the target of the cyberbullying incident. The incident was required to be simulated within one minute. Experimenters could appropriately respond to others’ reactions or questions. Third, participants’ responses to the cyberbullying incidents in the groups were observed and recorded three times.

For the first time, the experimenters recorded the data (e.g., group information and reactions of group members) within five minutes after presenting the cyberbullying incidents. If participants’ responses included non-text content (e.g., QQ emoji), they were recorded based on the original meaning of the content and the experimenters’ speculation. The responses that were not related to the cyberbullying incidents would not be recorded. The second record was performed two hours after the first record. The third record was carried out about 6–12 h after the second record. The information in these two recording times was similar to the first recording.

The Ethics Committee of the authors’ university approved the present study. No relationship was established between the experimenters and the participants before the study began. Because the present study aimed to obtain participants’ actual behavioral responses to cyberbullying, the informed consent of the participants was not solicited before the experiment. After the data collection, the researchers explained the purpose and other information of the experiment to the QQ groups. They also ensured that the personal information of participants would be kept confidential and that the data would be used only for experimental research. The experimental data were collected from February 17 to March 2, 2020, lasting about two weeks.

#### Data analyses

All the participants’ relevant chat content was transcribed verbatim, and the pictures/emoji were recorded in text form and input into Excel 2016 and SPSS 21.0. The whole data were sorted out and analyzed by using Word 2016 and NVivo 11.0. The team inspected all the data and eliminated irrelevant information, such as the members’ responses suggesting that they had known that the cyberbullying incident was acted. Other analytical procedures were the same as in Study 1.

### Results

**Figure 1:**
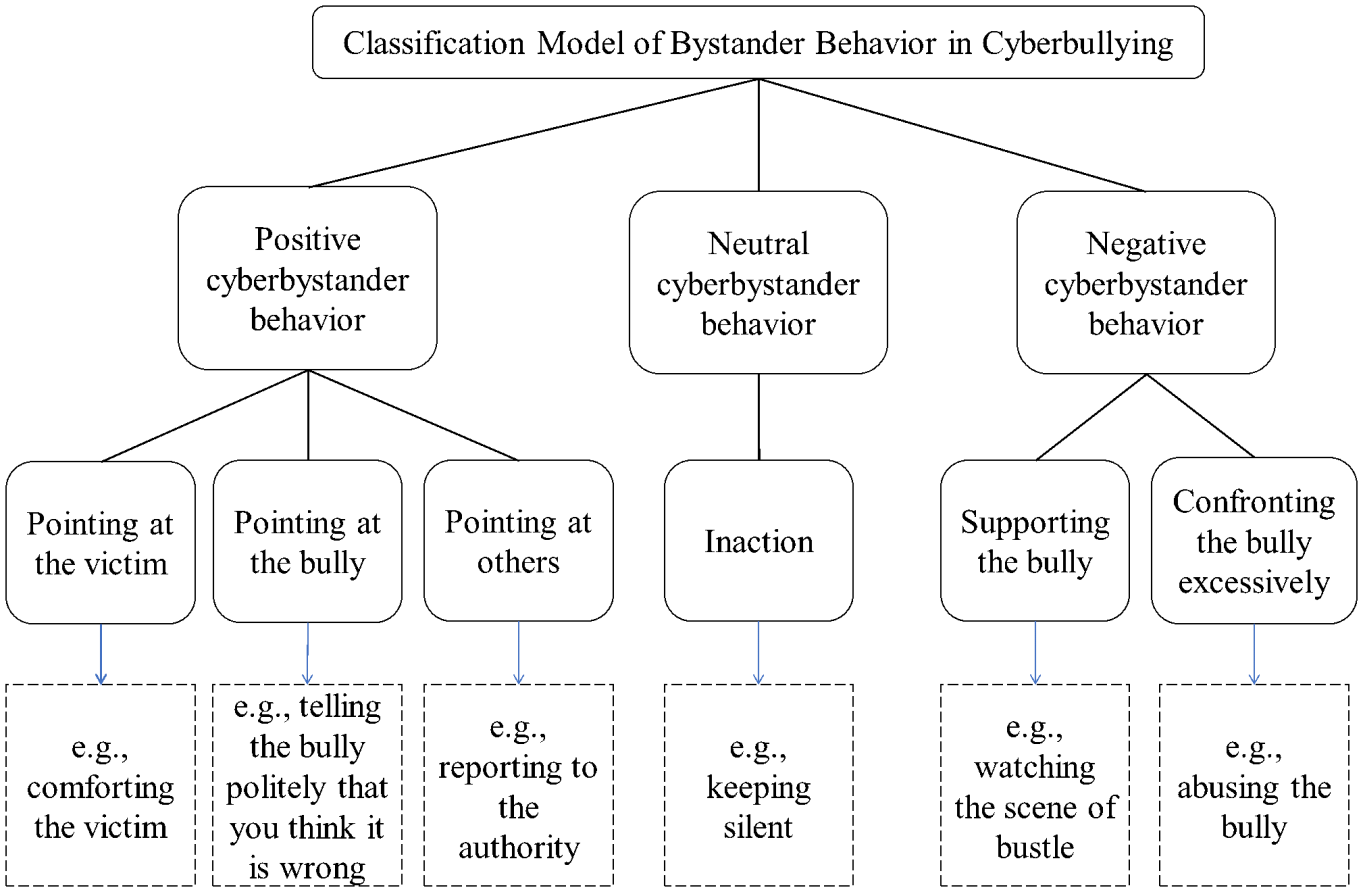
Classification Model of Bystander Behavior in Cyberbullying.

The results of the qualitative analysis of participants’ actual behavioral responses to cyberbullying incidents were displayed in Table 2. The classification model of bystander behavior in cyberbullying had three first-level factors (i.e., positive, neutral, and negative bystander behavior) and 6 sec-level factors (i.e., pointing at the victim, bully, and others, inaction, supporting the bully, and confronting the bully excessively). In the framework of the classification model, the actual behavioral responses had no big change compared to the behavioral intentions. However, there were some differences in the third-level factors. For instance, participants did not privately chat in the actual situation. Moreover, the inaction in the actual behavioral responses included not only doing nothing but also doing the useless thing. Combining the results of qualitative analyses obtained in Study 1 and Study 2, this study concluded with the following classification model (see Figure 1).

**Table 2:** Participants’ actual behavioral responses to cyberbullying incidents.

First-level codes	Second-level codes	Third-level codes	Examples
Positive cyberbystander behavior (196)	Pointing at the victim (100)	Supporting the victim (4)	Me too.	Comforting the victim (8)	I did not call you names. Do not worry.	Defending the victim (4)	Any question can be put forward for communication.	Encouraging the victim (3)	Come on!
	Pointing at the bully/confronting the bully moderately (80)	Expressing the speechlessness of the bully (37)	……	Telling the bully politely that you think it is wrong (14)	That’s not a very nice thing to say, is it?	Providing suggestions to the bully (8)	Mmmmm… Let us not get into the details.	Requiring the bully (10)	Please pay attention to your words.	Placating the bully (1)	We are all top students.	Breaking the ice (3)	Well, watch what you say. Maybe he is a super member.	Changing the subjects (7)	Forget it. Let us read books.
	Pointing at others (16)	Reporting to the authority (16)	Is the group leader here?
	Neutral cyberbystander behavior (>52/about 74,500)	Inaction (>52/about 74,500)	Not knowing the situation (38)	???	Express fear (14)	Frightened!	Keeping silent (…/about 74,490)	[Nothing sent.]
		Negative cyberbystander behavior (82)	Supporting the bully (46)	Praising the bully (7)	Awesome!	Watching the scene of bustle (31)	hahahaha	Encouraging the bully (3)	Bro, speak up.	Joining the bully (5)	Ugly people make more trouble.
			Confronting the bully excessively (36)	Taunting the bully (13)	Get over yourself.	Questioning rhetorically the bully (14)	It’s none of your business./How can you do that?	Abusing the bully (9)	Fuck off.

The numbers in brackets indicate the frequency of such actual behavioral responses.

On the whole, most participants showed neutral behaviors, followed by positive behaviors and negative behaviors. Specifically, the number of participants who showed overt neutral behavioral responses was low, but the number of participants who showed covert neutral behavioral responses (i.e., doing nothing) was high. The positive behaviors that most participants responded to were pointing at the victim and pointing at the bully. The former included helping the victim (e.g., responding to the victim and offering suggestions) and comforting the victim. The latter included expressing nothing to say to the bully and telling the bully politely that you think it is wrong. Besides this, participants’ negative behavioral responses focused on confronting the bully excessively and supporting the bully by watching the scene of bustle.

### Discussion

The proportion of positive behaviors in actual behavioral responses is lower than in behavioral intentions, which might suggest the effect of social desirability on participants’ behavioral intentions in Study 1. Participants tend to rarely engage in private chat and take a wait-and-see attitude in real situations in Study 2. Nevertheless, in Study 1, many participants are inclined to choose to chat with the bully or victim in private. It is probably because participants are less familiar with both the bully and victim. The inference can be evidenced through the statements of participants in Study 1. For example, some participants say that they are more likely to choose private chats with acquaintances.

In the real context, participants are more likely to exhibit neutral behavior (i.e., inaction). Their inaction may be explained by the Information Overload Theory (Hamby et al., 2014). The speed of information reflection by receivers is far slower than the speed of information transmission, for social information is far beyond the range that can be accepted, processed, or effectively utilized by individuals or systems. A great deal of irrelevant, useless, and redundant data information seriously interferes with the audiences’ selection of the accuracy of relevant and useful information. Hence, online bystanders, numbed by the amount of information that they receive every day, become silent.

Results show that participants rarely show direct support for the bully and harm to the victim. This may be because of the following reasons. First, the Internet is a public place and is governed by a system of rules and morals. Direct support for bullying will be sanctioned by morality. Second, in terms of biological evolution, humans themselves are relatively weak, and they rely on mutual help between groups, especially to help the weak survive. Therefore, people tend to sympathize with the weak. Besides, the weak are generally not aggressive and are not easier to stimulate others’ internal defense mechanisms, while the strong behave more aggressively and are difficult to be accepted by others. In cyberbullying incidents, the bully is stronger than the victim, so bystanders are probable to sympathize with the victim than join in the bullying. Third, China is a collectivist country. It is less likely for bystanders to initially harm one person in a group, especially if the other members of a group are unfamiliar with each other.

However, participants tend to behave in some relatively indirect forms of negative behavior, such as watching the scene of bustle and taunting the bully. These behaviors are not directly malicious toward the victim but may indirectly harm them. Because bystanders may not realize that these behaviors are negative but instead perceives them as positive. For instance, it is human nature to watch the scene of bustle, and people usually believe that they are just looking. Nevertheless, differing from the inaction, bystanders’ reactions when watching often reflect their interest in the incident and can easily satisfy the bully’s desire to get attention (Jones, 2014). Their role is similar to reinforcers for the bully. Taunting the bully can intensify the bully’s negative emotions, which is likely to make the bully harm the victim more severely. Or when bystanders aggress excessively toward the bully, it can easily turn into a new round of cyberbullying against the original bully.

## General discussion

Second-level factors are an important feature of this classification model, which will be specifically explained. The 3 sec-level factors of bystander positive behaviors are mainly classified according to the target people, which generates the different effects of the intervention. Specifically, the behavior of pointing at the victim usually struggles to have a significant intervention effect on the cyberbullying incidents but can alleviate the harm of cyberbullying incidents to the victim to a certain extent. The behaviors of pointing at the bully and others enable curbing the deterioration and extension of incidents to protect the victim effectively. Seeking help from authorities such as administrators could stop the incidents more rapidly. Previous research attaches importance to the study of positive behaviors, but relatively neglects the study of positive behavior classification (e.g., Olenik-Shemesh et al., 2015; DeSmet et al., 2016). Desmet et al. (2014) found that positive and negative behaviors often occurred together. Therefore, the present study further subdivides positive behaviors to separate the latent negative behaviors among them (i.e., confronting the bully excessively). Intervention for positive behaviors can also be more targeted according to the classification of the objects.

The 2 sec-level factors of bystander negative behaviors are classified primarily based on whether the starting point of the bystander’s behavior is to protect the victim. The behaviors of supporting the bully will encourage the bully’s arrogance, increase the spread of cyberbullying incidents, and cause the victim to suffer more serious injuries. The behavior of confronting the bully excessively usually provokes the bully’s anger and creates conflict between the bully and bystanders, leading to a vicious cycle of cyberbullying incidents. Previous research on the classification typically considers negative behavior as joining in the bullying (e.g., Van Cleemput et al., 2014), or divides it into assisting and reinforcing (e.g., Song and Oh, 2018). The present study categorizes behaviors like reinforcing and assisting as supporting the bully. Because they are harder to be distinguished in cyberbullying. For instance, the behavior of forwarding the bully’s comments is both reinforcing and assisting. In addition, in the results of the two qualitative studies, fewer people will join in the bullying directly, and more people tend to exhibit the behavior of confronting the bully excessively that is often been ignored by previous studies. Taking the influence of traditional Chinese culture, Chinese people may prefer to be in a united and harmonious group. If someone attempts to break this harmony, it is easy for others to resist. While if this resistance is too aggressive, it often does not ease the conflict but worsens it. Therefore, it is necessary to classify the excessively confronting the bully separately and study it further.

The neutral behavior of inaction in the study includes not only the traditional sense of no action but also the act of doing something which effect is the same as doing nothing. On the one hand, inaction may to some extent defeat the goal of bullies, which is to draw attention (Jones, 2014). In other words, if cyberbullying is ignored, it may disappear. This behavior can also be regarded as positive for the victim. Because harmful actions and words do not continue to spread, which stops the continuation of cyberbullying incidents (Barlińska et al., 2013). On the other hand, inaction enables bullies to believe that their behaviors are recognized and accepted by others and thus may abet cyberbullying (Kowalski et al., 2014; DeSmet et al., 2016). This behavior also harms the victim because they may feel “borderline insecure” or think “everyone agrees,” leading to extreme effects such as suicide (Jones, 2014).

In the two qualitative studies, most participants show neutral behavior, with a great number of those keeping silent. There may be several reasons. First, although participants are online, they may not see the group messages in time or miss these messages due to too much information received (Hamby et al., 2014). Second, when participants see the messages, they do not know how to solve the problem. Hence, they choose to keep silent and wait to see what others do. The inference can be evidenced through the statements of participants in Study 1. Third, participants choose to ignore the cyberbullying incident with various concerns such as fear of retaliation from the bully or thinking that the incident has nothing to do with them. To sum up, the influence of neutral bystander behavior on cyberbullying incidents is more complex. Inaction should not be simply classified as positive or negative behavior but as a separate category. Future studies can further subdivide the neutral classification of bystander behavior in cyberbullying from the perspective of the reasons for bystander inaction.

The main differences between the behavior of pointing at the bully (i.e., confronting the bully moderately) and the behavior of confronting the bully excessively are whether bystanders confront the bully intellectually or emotionally and whether their behaviors cause the bully to retaliate or not. In the behavior of pointing at the bully, bystanders stand on the side of justice, rules, and morality. They express their moral outrage by appropriate approaches and impose moral sanctions on the cyberbully to make him feel guilty and ashamed, and then prevent the continuation and recurrence of cyberbullying incidents. In the behavior of confronting the bully excessively, bystanders vent their dissatisfaction and hatred, which is more likely to arouse the resistance and anger of the cyberbully and worsen the cyberbullying incidents. This behavior is an excessive expression of moral outrage, akin to moral kidnapping. In addition, it is appropriate for one bystander to express moral outrage against the bully, but when many people go along with it so that viral outrage forms, others may, in turn, sympathize with the bully and condemn the bystanders (Sawaoka and Monin, 2018). Therefore, in the behavior of confronting the bully excessively, the bully is easily transformed into the victim, while bystanders are easily transformed into the bully in cyberbullying incidents.

### Implications and limitations

This model could help researchers to develop more effective intervention approaches for each bystander behavior category to achieve the ultimate goal of cyberbullying intervention. First, education about prevention and supportive approaches like the HAHASO program (Mishna et al., 2011) can enhance bystanders’ positive behavioral responses when allowed to take on a supportive role (Mishna et al., 2011; Wong-Lo et al., 2011). Also, relevant education can help them realize that confronting the bully excessively is not a good way. Second, Wakslak et al. (2007) believe that moral outrage is an important motivation for action to help the weak. It could form certain moral constraints not only for themselves but also for others. Bystanders with inaction and excessively confronting the bully could be cultivated appropriate moral outrage by enhancing justice sensitivity. The moral outrage exhibited by people with high justice sensitivity is the emotion that directs at immoral and unjust acts rather than anger mixed with egoism (Rothschild and Keefer, 2018), which may not produce the behavior of confronting the bully excessively. Third, strategies such as using online privacy protection and anonymous reporting can provide additional reassurance to bystanders who may do nothing for fearing retaliation (Mickie and Lyndal, 2014), which may increase the likelihood of helping the victim as inaction bystanders. Fourth, Levine and Crowther (2008) found that social group membership is more important than group size in predicting bystanders’ intervention in cyberbullying incidents. Therefore, it is necessary to create a harmonious group atmosphere, which could make bystanders more likely to help the victim and less likely to join in the bullying.

It should be recognized that this study also has some limitations. First, Study 1 uses a situational simulation with static screenshots, in which participants may have a weak sense of substitution and be susceptible to social desirability. Therefore, their behavioral intentions collected may not be their true thoughts. To address this problem, the Marlowe-Crowne Social Desirability Scale (MCSD, Crowne and Marlowe, 1960; Wei et al., 2015) could be added at the end of the questionnaire. When collating the data, the data with high social desirability should be singled out for separate analysis. Second, the content of the eight types of cyberbullying incidents should be improved. The contents of them are a little unrealistic or less common, so it’s easy for group members to see through them and not respond. Third, in the experimental study of bystanders’ actual behavioral responses, participants may be familiar with each other in the chat with a small number of members, while they may be more unfamiliar with each other in the chat with a large number of members. The relationships among participants are likely to affect their behavioral responses, and the influence of identity and relationship on bystander behavioral responses in cyberbullying could be further studied in future research.

There are some details that the model in the study could be further studied. First, further research is needed on the extent to which the three categories of positive cyberbystander behavior. It may be useful in studying more effective measures of protecting the victim. Second, the criteria for distinguishing between pointing at the bully (i.e., confronting the bully moderately) and confronting the bully excessively need to be further refined to make them discriminate accurately. Third, the classification of neutral cyberbystander behavior needs to be further subdivided. For example, their behaviors of inaction could be classified according to their motivations.

## Conclusion

Due to the large number of bystanders in cyberbullying, their behavioral responses play an important role in the development of cyberbullying incidents and this influence is generally direct and effective. This study constructs a classification model of bystanders’ behavior in cyberbullying incidents through depth analysis of their behavioral intentions and actual behavioral responses in two qualitative studies. The classification model includes positive bystander behavior (i.e., pointing at the victim, bully, and others), neutral bystander behavior (i.e., inaction), and negative bystander behavior (i.e., supporting and excessively confronting the bully). The classification model has significant value to the research on bystander behavior in cyberbullying, including labeling confronting the bully moderately (i.e., pointing at the bully) as positive bystander behavior and labeling confronting the bully excessively as negative bystander behavior. This study is conducted from the perspective of cyberbystander behavior classification and the results have a certain reference value for cyberbullying intervention. Future studies could further distinguish these categories of behaviors in terms of causes and intervention measures.

## Data availability statement

The raw data supporting the conclusions of this article will be made available by the authors, without undue reservation.

## Ethics statement

The studies involving human participants were reviewed and approved by Institute of Psychological and Brain Sciences, Zhejiang Normal University. Written informed consent for participation was not required for this study in accordance with the national legislation and the institutional requirements.

## Author contributions

KR: Conceptualization and writing–original draft. XC: investigation, review and editing, and funding acquisition. YZ: review and editing. All authors contributed to the article and approved the submitted version.

## Funding

The study was supported by The National Social Science Foundation of China [Project No. CBA210234].

## Conflict of interest

The authors declare that the research was conducted in the absence of any commercial or financial relationships that could be construed as a potential conflict of interest.

## Publisher’s note

All claims expressed in this article are solely those of the authors and do not necessarily represent those of their affiliated organizations, or those of the publisher, the editors and the reviewers. Any product that may be evaluated in this article, or claim that may be made by its manufacturer, is not guaranteed or endorsed by the publisher.
